# p53, a potential predictor of *Helicobacter pylori* infection-associated gastric carcinogenesis?

**DOI:** 10.18632/oncotarget.11414

**Published:** 2016-08-19

**Authors:** Nianshuang Li, Chuan Xie, Nong-Hua Lu

**Affiliations:** ^1^ Department of Gastroenterology, The First Affiliated Hospital of Nanchang University, Nanchang, Jiangxi, China

**Keywords:** Helicobacter pylori, p53, tumourigenesis

## Abstract

*Helicobacter pylori* (*H. pylori*) is an ancient and persistent inhabitant of the human stomach that is closely linked to the development of gastric cancer (GC). . Emerging evidence suggests that *H. pylori* strain interactions with gastric epithelial cells subvert the best- characterized p53 tumour suppressor pathway. A high prevalence of p53 mutations is related to *H. pylori* infection. *H. pylori* also accelerates p53 protein degradation by disturbing the MDM2-P53 feedback loop. Additionally, *H. pylori* triggers the alteration of other p53 isoforms. Dysregulation of p53 by *H. pylori* infection contributes to gastric carcinogenesis by mediating cell proliferation and apoptosis. This review focuses on the regulation of p53 in *H. pylori* infection-associated GC.

## INTRODUCTION

*Helicobacter pylori* infection is a strong risk factor for gastric cancer (GC). The International Agency for Research on Cancer (IARC) classified this bacterium as a Group I carcinogen. Epidemiological studies indicate that approximately 50% of the global population is infected with *H. pylori*, which is primarily acquired during childhood [1-3]. *H. pylori* uses various virulence factors in gastric epithelial cells to target different signalling pathways that regulate specialized survival programs, such as the cell cycle and apoptosis. Vacuolating cytotoxin gene (vacA) and cag pathogenicity island (cagPAI) are the best characterized virulence factors [4]. cagPAI encodes a type IV secretion system (T4SS) that delivers the oncoprotein CagA into host cells.

Chronic inflammation results from the virulence factor CagA and is recognized as the underlying mechanism of *H. pylori*-associated gastric carcinogenesis [5]. Accumulating data demonstrate that CagA injection into host cells stimulates the recruitment of inflammatory cells and the production of cytokines, including interleukin (IL)-1β and IL-8, as well as the activation of Wnt/β-catenin signalling via the nuclear accumulation of beta-catenin in epithelial cells [6-8]. *H. pylori* also promotes PI3K/AKT, mTOR, and p38/MAPK signalling, and these oncogenic signalling pathways alter cellular oncogenes and tumour suppressors. *H. pylori* also disrupts cell-cell junctions and cell polarity via inhibition of the PAR-1/MARK complex [9, 10]. *H. pylori* infection causes DNA double-strand breaks (DSBs) and genomic instability via the generation of reactive oxygen species (ROS) and reactive nitrogen species (RNS) [11, 12]. Together, recent studies have shown that the regulation of p53 is involved in gastric carcinogenesis caused by *H. pylori* infection.

The pathogenic bacterium *H. pylori* modulates the host p53 pathway by controlling various cellular stresses, including DNA damage, ROS and RNS production, and abnormal oncogene activation. Frequent p53 gene mutations are also detected in gastric pre-neoplastic lesions, which more broadly suggests an association between p53 and disturbances in cell proliferation and apoptosis in gastric epithelial cells infected with *H. pylori*. This review focuses on *H. pylori* infection-mediated p53 regulation because of the central role of p53 in *H. pylori*-induced gastric carcinogenesis. Thus, p53 may serve as a novel biomarker and therapeutic target for GC.

## TUMOUR SUPPRESSOR P53

The human tumour suppressor gene p53, also known as TP53, was first identified in 1979 as a cellular protein bound to immunoprecipitates of the SV40 LT protein in SV40 tumour virus-transformed cells [13]. The p53 is present at extremely low levels in normal cells, but these levels increase significantly in response to DNA damage or other types of cellular stress [14]. In particular, DNA damage induces the stabilization and nuclear accumulation of p53 via the DNA damage signalling pathway, which involves sensor kinases such as ataxia-telangiectasia mutated (ATM) and ataxia-telangiectasia and Rad3-related (ATR) [15, 16].

**Figure 1:**
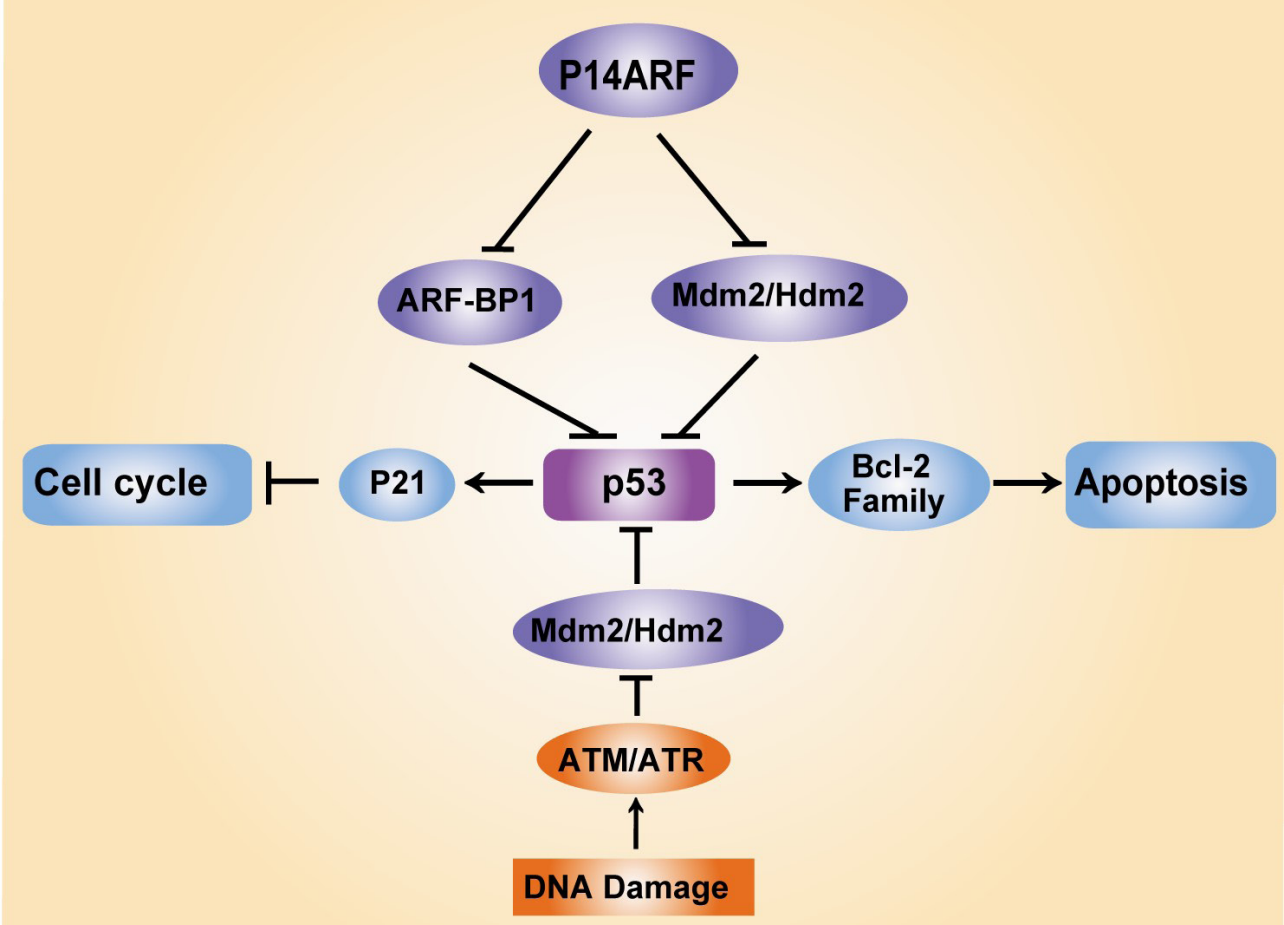
Regulation of p53 and its key regulators in response to DNA damage DNA damage triggers the activation of the tumour suppressor protein p53 *via* ataxia-telangiectasia mutated (ATM) and ataxia-telangiectasia and Rad3-related (ATR) protein kinase-dependent pathways, which leads to cell cycle arrest *via* the induction of the cyclin-dependent kinase (CDK) inhibitor p21 and cell apoptosis *via* mediation of Bcl-2 family expression. MDM2 (also known as HDM2) and ARF-BP1 act as E3 ubiquitin ligases that target p53 proteasomal degradation, which ensures low p53 levels in normal and unstressed cells. Notably, the tumour suppressor p14ARF protects p53 protein stability *via* inhibition of MDM2/HDM2 and ARFBP1.

p53 is known as the “guardian of the genome” because it repairs DNA and maintains genomic stability after DNA damage. The ubiquitin ligases murine double minute (MDM2, also known as HDM2) and ARF-BP1 are crucial negative regulators of p53 that drive its rapid degradation [17, 18]. Notably, p14ARF regulates the transcriptional activity of p53 by antagonizing MDM2. The p53 protein functions as a transcription factor that promotes cell cycle arrest at the G1 phase *via* upregulation of the expression of an inhibitor of cyclin-dependent kinase, p21. p53 also induces cell apoptosis *via* two apoptotic pathways, the mitochondrial pathway, which mediates the expression of B-cell lymphoma 2 (Bcl-2) family members, and the death receptor pathway, which triggers activation of the caspase cascade [19-21] (Figure 1). Indeed, approximately 50% of all cancers exhibit a mutation in p53 [22]. *In vivo* assays have shown that mice expressing a mutant p53 protein exhibit more aggressive and metastatic tumours compared to p53 wild-type (WT-p53) mice [23]. These findings suggest that the mutation of p53 during tumourigenesis represents an essential step in the switch to aggressive tumour development.

## EXPRESSION OF P53 IN *H. PYLORI*-INFECTED PATIENTS WITH GASTRIC CARCINOGENESIS

GC is divided into three pathological variants: intestinal type, diffuse type and mixed type [24, 25]. *H. pylori* has been identified as the strongest risk factor of intestinal type GC, which may progress from atrophy gastritis to intestinal metaplasia, dysplasia and eventually carcinoma [26]. Some investigations have detected p53 expression in the gastric mucosa of patients with pre-neoplastic lesions infected with *H. pylori*, including atrophy and intestinal metaplasia [27, 28]. Immunohistochemical analyses of p53 in CagA-positive *H. pylori* gastritis patients prior to gastric metaplastic/dysplastic changes have revealed increased p53 expression but decreased apoptosis, and one possible cause for this association is the detection of mutant p53 protein [29]. p53, p21 and Ki-67 expression were examined using immunohistochemical staining in volunteers from the general Spanish population. The subjects with *H. pylori* infection exhibited significantly higher p53 levels and mucosal proliferative activity compared to the uninfected subjects, and this association was primarily related to cardia-type mucosa rather than corpus [30]. A 3-month prospective study was performed to classify the relationship between *H. pylori* infection and p53 in patients with intestinal metaplasia, and the results indicated an obvious association of *H. pylori* infection with p53 expression [31].

Wild-type p53 protein is relatively unstable and generally not detectable. In contrast, mutant p53 exhibits a much longer half-life, and immunohistochemical staining of p53 is commonly used as a surrogate for mutational analyses [32]. Gastric biopsy samples from gastritis, intestinal metaplasia and GC patients were collected to measure p53 expression levels in gastric carcinogenesis related to *H. pylori* infection. p53 protein exhibited a significantly higher degree of staining in *H. pylori*-positive GC samples; specifically, p53-positive cells were identified in 0% of control samples, 0% of gastritis samples, 13.3% of intestinal metaplasia samples and 43% of GC samples [33, 34]. These results suggest that alteration of p53 may be an early event in *H. pylori*-associated gastric carcinogenesis.

## P53 GENE MUTATIONS RELATED TO *H. PYLORI* INFECTION

Inactivation of the tumour suppressor p53 is a hallmark of tumourigenic changes. Accordingly, the p53 gene is aberrantly expressed in nearly half of all human cancer cell genomes. Mutant p53 proteins acquire tumour-promoting functions that enhance cell growth, proliferation, survival, and motility [35, 36] p53 mutations are generally divided into two categories: DNA-interacting mutants that directly alter amino acids and structural mutants that lead to the unfolding of p53 protein, which is critical for tumours to gain tumour-associated functional phenotypes [37, 38]. Notably, the primary alterations of p53 occur primarily in highly conserved domains, especially amino acid substitutions, including R175, G245, R248, R249, R273 and R282. Some mutant p53 proteins that recognize a unique response element acquire properties of an oncogenic transcription factor. Mutant p53 was experimentally demonstrated to deregulate checkpoints of cell cycle responses to the induction of DNA damage, which accelerated tumour progression via the promotion of genomic instability, cell proliferation and invasion [39, 40].

Numerous studies have shown that *H. pylori* infection is associated with a higher prevalence of p53 mutation. Indeed, an increasing frequency of p53 alterations occurs in neoplastic gastric lesions ranging from gastritis through dysplasia, intestinal metaplasia ( IM ) and advanced GC [41-43]. *H. pylori* is the strongest risk factor for GC, and this infection is known to enhance p53 mutagenesis. Lopez-Saez et al. found that 81% of the *H. pylori*-associated GC deaths in Spain were associated with increased mutant p53 protein compared to 11% in the *H. pylori*-negative group[44]. *H. pylori* infection may also contribute to the development of gastric adenocarcinoma by elevating mutant p53 protein [45]. The accumulation of mutant p53 may be attributed to the production of free radicals, such as ROS and RNS [46]. The combination of whole-exome sequencing with deep sequencing revealed that C:G>T:A transitions at XpCpG trinucleotides were the most common accumulation pattern of TP53. A total of 105 GC specimens from a high-risk area in Italy were examined for the prevalence of p53 mutations[47]. Notably, an association of *H. pylori* infection with p53 mutations was also apparent at CpG sites[47].

Mutations of p53 in exon 5-8 nucleotide sequences have also been widely studied in primary gastric adenocarcinoma with *H. pylori* infection [48]. Nucleotide sequencing analyses demonstrated the induction of several point mutations in exons 5, 6, 7 and 8 of the p53 gene in *H. pylori*-infected monkeys compared to *H. pylori*-uninfected monkeys, and p53 gene mutations were closely related to the degree of gastric mucosa atrophy [49]. Alterations in exons 5-8 of the p53 gene were also observed in *H. pylori*-positive patients with GC and peptic ulcer disease (PUD) [50, 51]. The well-characterized factor CagA is an important virulence determinant in the gastric carcinogenesis of *H. pylori* infection. Notably, the p53 gene is more likely to harbour p53 mutations in gastric tumours with CagA-positive *H. pylori* strains. Recent studies suggest that gastric epithelial cells infected with CagA-positive *H. pylori* induce the expression of cytidine deaminase (AID), which may be a mechanism of p53 mutation accumulation during *H. pylori*-associated gastric carcinogenesis [28, 52].

## P53 CODON 72 POLYMORPHISMS

Several polymorphic sites in the tumour suppressor p53 are closely associated with various cancer risks. The codon 72 polymorphism that produces variant genotypes with arginine (Arg) or proline (Pro) is a frequently reported polymorphism site [53-55]. Hiyama et al. demonstrated that Pro/Pro genotypes at the p53 codon 72 polymorphism contribute to susceptibility to diffuse-type gastric carcinoma in patients with chronic gastritis [56]. One study reported that the polymorphism is related to lower TP53 transcriptional levels [55]. The distribution of the p53 codon 72 polymorphism varies in different regions worldwide. PCR-Taqman assays identified the Arg/Pro genotype of the p53 codon 72 polymorphism in 140 patients with gastric carcinoma from the GanSu province in China, where the GC incidence is high, and the p53 codon 72 Pro carrier genotype with *H. pylori* infection was consistently higher in GC groups than controls [57]. The p53 Pro/Arg and p53 Arg/Arg single-nucleotide polymorphism (SNP) variants were also significantly more common than the p53 Pro/Pro variant in a population from North India, which exhibits a high prevalence of *H. pylori* infection but low GC incidence. Therefore, the Arg or Pro/Arg polymorphism may underlie these differences [58].

## REGULATION OF THE P53-MDM2 FEEDBACK LOOP

The p53 pathway is extremely sensitive to DNA double-strand breaks (DSBs) and single-stranded DNA (ssDNA) gaps, which are created by a variety of DNA-damaging agents, including ionizing radiation, chemotherapeutic drugs and ultraviolet (UV) radiation [59, 60]. The transcription-activating powers of p53 depend on the regulation of ATM and ATR protein kinases, which transfer the p53 signals to cell cycle checkpoint 1 (Chk1) and cell cycle checkpoint 2 (Chk2) kinases and lead to the phosphorylation of p53 to protect it from destruction by the specific E3 ubiquitin ligase murine double minute 2 (MDM2) [61, 62].

The MDM2 protein has emerged as a crucial regulator of p53 tumour suppressor function. MDM2 binding to p53 immediately induces proteasome degradation of p53 and blocks p53-driven transcription [17, 63]. The underlying molecular mechanism of MDM2 regulation of p53 degradation has been elucidated; MDM2 binds to the N terminus of p53 and promotes its export from the nucleus to cytoplasm, which results in its rapid degradation in cytoplasmic proteasomes [64]. p53 elements are located in the promoter of MDM2, and p53 induces the expression of MDM2. Therefore, a negative feedback loop exists between p53 and MDM2 that ensures low cellular p53 levels in normal and unstressed cells [65].

The amplification and overexpression of MDM2 are frequently observed in a variety of human cancers [66, 67], and *H. pylori* also suppresses p53 through multiple mechanisms [68]. This bacterium contributes to proteasomal degradation of p53 by increasing the phosphorylation of the E3 ubiquitin ligase MDM2 [69, 70]. Indeed, elevated levels of MDM2 are found in *H. pylori*-infected intestinal metaplasia and GC [71]. p53 was also shown to be upregulated in a bimodal fashion over time in Mongolian gerbils, which are the most suitable laboratory animal model for the study of *H. pylori* infection [69, 72]. Acute accumulation of p53 was observed in response to cellular stressors after initial *H. pylori* infection, with subsequent rapid downregulation of p53 in the gastric mucosa; a second peak of p53 occurred after gastritis development [73]. These studies suggest that alterations in p53 expression profiles depend on the duration of *H. pylori* infection.

## UPSTREAM REGULATORS OF THE MDM2-P53 FEEDBACK LOOP IN THE PATHOGENESIS OF *H. PYLORI*

*H. pylori* infection increases the phosphorylation and activation of AKT and MDM2 to downregulate p53, which results in the inactivation of p53 by increased ubiquitination and proteasome degradation [74, 75]. Our previous studies investigated the correlation between protein expression in the AKT-MDM2-p53 pathway and *H. pylori* infection in chronic non-atrophic gastritis (CNAG), metaplastic atrophy (MA), gastric dysplasia (Dys) and GC patients. Notably, higher levels of mutant p53 and MDM2 were observed in *H. pylori*-positive patients with MA and Dys, and the expression of phosphorylated AKT was upregulated in CNAG patients with *H. pylori* infection. The expression of pAKT and MDM2 was also significantly increased after *H. pylori* strains were co-cultured with GES-1 gastric epithelial cells, which also supports the degradation of p53 [76, 77]. Inhibition of MDM2 activity using siRNA or the inhibitor Nutlin3 in AGS human GC cells clearly inhibited the downregulation of *H. pylori*-induced p53. Notably, the bacterial protein CagA plays an important role in the alteration of p53 protein, as ectopic expression of CagA is sufficient to enhance AKT and MDM2 activity and induce p53 degradation [73, 77].

ERK kinase interactions with HDM2 are responsible for p53 activity in *H. pylori*-infected cells [78], and experimental inhibition of ERK/MDM2 interactions was shown to rescue p53 activity and dampen damaged cell survival [79]. The intercellular junction protein E-cadherin, which maintains the epithelial barrier, was also upregulated in these gastric epithelial cells. AKT and ERK contribute to MDM2 activation in the setting of *H. pylori* infection, which leads to degradation of the p53 protein. Notably, p53 was dynamically altered in gastric epithelial cells co-cultured with *H. pylori*, which suggests a crucial role of the p53-MDM2 feedback loop [80].

P14ARF inhibits cell proliferation in a p53-dependent manner. Specifically, p14ARF segregates MDM2 in the nucleolus and antagonizes the E3 ubiquitin ligase-mediated p53 protein degradation [81, 82]. *H. pylori* contains high levels of bacterial CagA protein, which rapidly induce p53 degradation, although p53 is protected by thetumour suppressor p14ARF. The oncogenic strain 7.13 of *H. pylori* efficiently induces gastric dysplasia and adenocarcinoma, and this strain was significantly more potent in attenuating the expression of p14ARF and p53 compared to the non-tumourigenic strain B128. These results were confirmed in gastric epithelial cells by the ectopic expression of protein or the downregulation of ARF using shRNA [83]. The p14ARF gene promoter is frequently methylated during the development of GC, and the p53 protein is rapidly degraded as a result[84].

## OTHER P53 ISOFORMS REGULATED BY *H. PYLORI* INFECTION

p63 and p73 are homologues of the p53 gene [85], and the high sequence similarity of their DNA-binding domain allows p63 and p73 to regulate p53-dependent cell cycle and apoptosis in response to DNA damage [86]. The P1 and P2 promoters of p63 and p73 promote the expression of a variety of protein isoforms, including the TA isoforms (TAp63 and TAp73) with an N-terminal transactivation domain (TAD) and the ΔN isoforms with diametrically opposed functions (ΔNp63 and ΔNp73) without the TAD [87, 88]. Elevated levels of p73 have been reported in *H. pylori*-positive human and murine tissues, and *in vitro* assays have demonstrated the role of p73 in the pathogenesis of *H. pylori* infection. AGS cell lines co-cultured with *H. pylori* exhibited significantly increased TAp73 expression, while ΔNp63 expression was suppressed. These findings were also confirmed using dominant-negative p73 mutants[89].

Δ133p53 is a truncated p53 isoform that is altered after *H. pylori* infection. The alternative promoter of the p53 gene, upstream of exon 1, or an internal promoter located in intron 4 results in an N-terminally truncated p53 protein initiated at codon 133 (Δ133p53) [90]. Growing evidence demonstrates that Δ133p53 is overexpressed in several tumour types [91, 92]. Wei et al. demonstrated that *H. pylori* infection induced Δ133p53 expression in AGS and SNU-1 gastric epithelial cells, and these findings were also observed in Mongolian gerbils. These cell lines were transfected with the endogenous Δ133p53 isoform shRNA plasmid, and the Δ133p53 isoforms affected the activity of p53 and p73, leading to the upregulation of the p53 target proteins p21 and phorbol-12-myristate-13-acetate-induced protein 1 (PMAIP1; also known as NOXA). Mechanistically, the activator protein-1 (AP-1) transcriptional complex, which is composed of c-Fos and c-Jun proteins, is also involved in the regulation of Δ133p53 in *H. pylori* cells [93, 94]. This finding is consistent with previous studies showing that *H. pylori* accelerated the progression to gastric intraepithelial neoplasia via the upregulation of AP-1 and NF-κB activity [95]. Notably, cagPAI is required for the regulation of p53 isoforms by *H. pylori*.

The p53 14-3-3 protein isoform binds to the regulatory C-terminal domain of p53, and this binding is subverted in *H. pylori*-associated gastritis and GC [96]. The p53 14-3-3 proteins are a class of adaptor proteins with seven isoforms (β, γ, ε, η, σ, τ, and ζ) that stabilize the tumour suppressor p53 and enhance its biological activity [96]. Proteasome and immunohistochemistry analyses have demonstrated that AGS cell lines show reduced expression of 14-3-3σ and 14-3-3η [97]. In summary, these studies suggest that p53 isoforms represent novel components of the host-defence mechanism and play an important role in *H. pylori*-associated cell proliferation and apoptosis.

## REGULATION OF THE APOPTOSIS-STIMULATING PROTEIN OF P53 (ASPP) FAMILY IN *H. PYLORI*-RELATED CARCINOGENESIS

The ASPP family functions as a key regulator of p53, and it consists of three members: ASPP1, ASPP2, and iASPP [98]. ASPP1 and ASPP2 enhance the ability of p53 to stimulate the expression of pro-apoptotic factors, such as Bax and p53-upregulated modulator of apoptosis (PUMA) [99]. Conversely, iASPP is a key inhibitor of p53 [100]. The apoptosis activator ASPP2 was downregulated and the apoptosis suppressor iASPP was upregulated in *H. pylori*-positive gastric carcinoma and pre-cancerous lesion tissues, which suggests that the ASPP family plays an important role in the gastric carcinogenesis of *H. pylori* infection [101].

The cancer-associated effector of the *H. pylori* CagA protein binds to residues 448-692 and 693-918 of ASPP2 [102]. After translocation into host epithelial cells, CagA induces the relocalization of ASPP2 rather than its abnormal expression and hinders the interaction between ASPP2 and p53, which is responsible for *H. pylori*-induced degradation of p53 and resistance to the apoptotic response [103, 104]. Suppression of apoptosis then contributes to the development of gastric dysplasia and adenocarcinoma [105]. This finding may be in conflict with previous studies showing that *H. pylori* infection stimulates host gastric epithelial cell apoptosis. However, the effect of cell apoptosis may be associated with continued *H. pylori* colonization, and a variety of genes are known to regulate cell apoptosis. Together, these findings show that the *H. pylori* CagA protein interaction with ASPP2 subverts the function of ASPP2 and p53, which facilitates *H. pylori*-induced gastric carcinogenesis.

**Figure 2:**
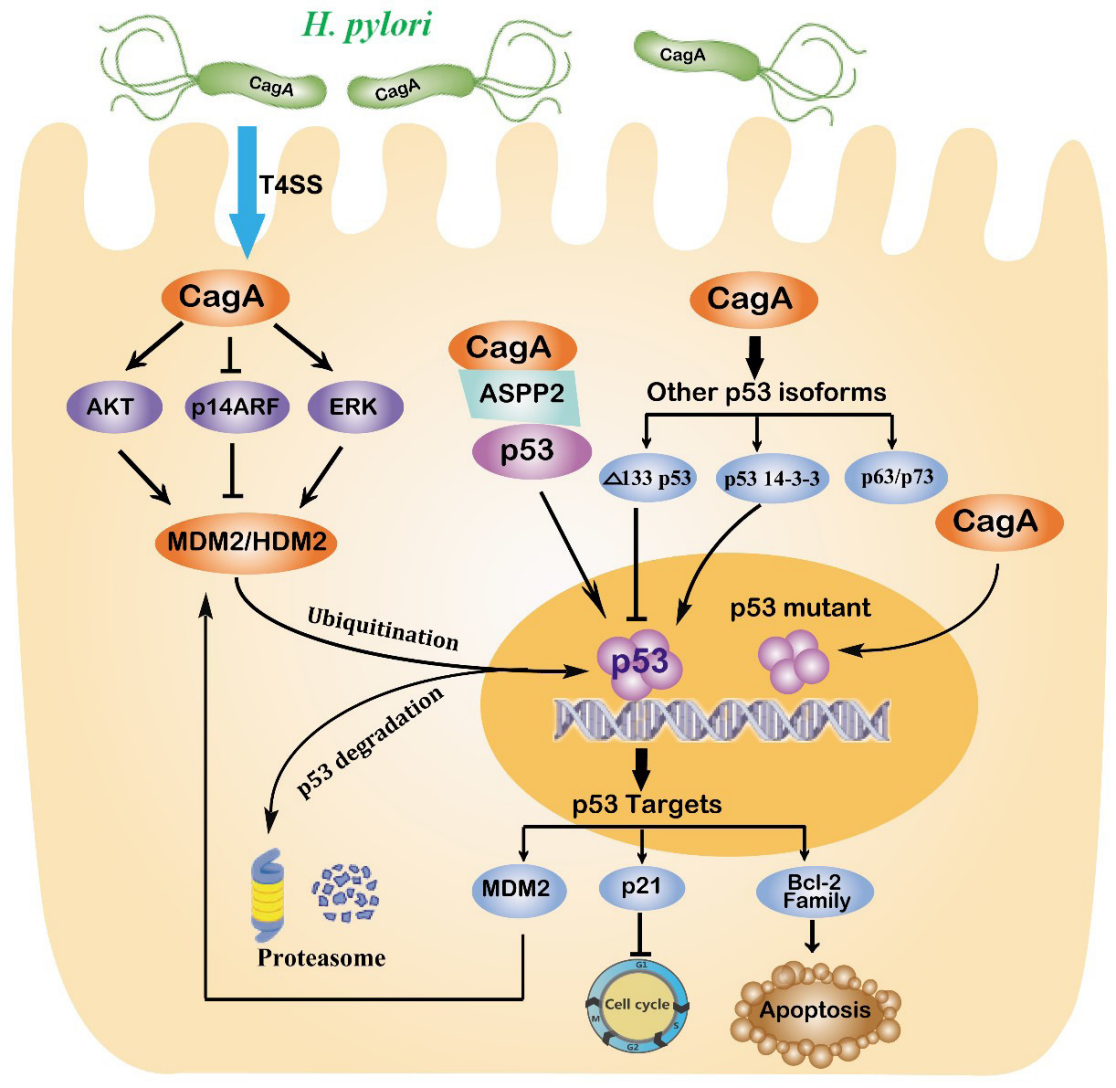
Regulation of p53 in gastric carcinogenesis induced by *H. pylori* *H. pylori* injects the oncoprotein CagA into gastric epithelial cells using a type IV secretion system (T4SS), which is responsible for alterations of the tumour suppressor p53. Chronic *H. pylori* infection may cause an accumulation of mutant p53. *H. pylori* also increases MDM2 levels *via* multiple mechanisms that target the proteasome-mediated degradation of p53. The CagA protein also binds to the apoptosis-stimulating protein of p53 (ASPP), which consequently hinders the interaction between ASPP2 and p53. Other isoforms of the p53 family, including p63, p73, Δ133p53 and p53 14-3-3 protein isoforms, are also involved in the regulation of p53 by *H. pylori*. In summary, long-term infection with *H. pylori* is responsible for reducing the levels of p53, which contributes to the development of gastric carcinoma.

## CONCLUSIONS

Infection with *H. pylori* leads to the aberrant expression of p53 protein through the induction of host DNA damage. The tumour suppressor p53 plays a major role in determining multiple signalling pathways and cellular responses. Chronic infection with the bacterial pathogen *H. pylori* may attenuate p53 levels via augmentation of p53 mutations, proteasome degradation and post-transcriptional modification. Furthermore, the expression of p53 transcriptional targets is inhibited and results in excessive proliferation and impaired apoptosis, which contributes to a greater risk of gastric carcinoma. Therefore, alterations of p53 may serve as a potential marker of *H. pylori*-associated gastric carcinogenesis.

## References

[1] Polk DB, Peek RM (2010). Helicobacter pylori: gastric cancer and beyond. Nature reviews Cancer.

[2] Salama NR, Hartung ML, Muller A (2013). Life in the human stomach: persistence strategies of the bacterial pathogen Helicobacter pylori. Nature reviews Microbiology.

[3] Amieva M, Peek RM (2015). Pathobiology of Helicobacter pylori-induced gastric cancer. Gastroenterology.

[4] Franco AT, Johnston E, Krishna U, Yamaoka Y, Israel DA, Nagy TA, Wroblewski LE, Piazuelo MB, Correa P, Peek RM (2008). Regulation of gastric carcinogenesis by Helicobacter pylori virulence factors. Cancer research.

[5] Suzuki M, Mimuro H, Kiga K, Fukumatsu M, Ishijima N, Morikawa H, Nagai S, Koyasu S, Gilman RH, Kersulyte D, Berg DE, Sasakawa C (2009). Helicobacter pylori CagA phosphorylation-independent function in epithelial proliferation and inflammation. Cell host & microbe.

[6] Franco AT, Israel DA, Washington MK, Krishna U, Fox JG, Rogers AB, Neish AS, Collier-Hyams L, Perez-Perez GI, Hatakeyama M, Whitehead R, Gaus K, O'Brien DP, Romero-Gallo J, Peek RM (2005). Activation of beta-catenin by carcinogenic Helicobacter pylori.

[7] Neal JT, Peterson TS, Kent ML, Guillemin K (2013). *H. pylori* virulence factor CagA increases intestinal cell proliferation by Wnt pathway activation in a transgenic zebrafish model. Disease models & mechanisms.

[8] Koch M, Mollenkopf HJ, Meyer TF (2016). Macrophages recognize the Helicobacter pylori type IV secretion system in the absence of toll-like receptor signalling. Cellular microbiology.

[9] Saadat I, Higashi H, Obuse C, Umeda M, Murata-Kamiya N, Saito Y, Lu H, Ohnishi N, Azuma T, Suzuki A, Ohno S, Hatakeyama M (2007). Helicobacter pylori CagA targets PAR1/MARK kinase to disrupt epithelial cell polarity. Nature.

[10] Nesic D, Miller MC, Quinkert ZT, Stein M, Chait BT, Stebbins CE (2010). Helicobacter pylori CagA inhibits PAR1-MARK family kinases by mimicking host substrates. Nature structural & molecular biology.

[11] Toller IM, Neelsen KJ, Steger M, Hartung ML, Hottiger MO, Stucki M, Kalali B, Gerhard M, Sartori AA, Lopes M, Muller A (2011). Carcinogenic bacterial pathogen Helicobacter pylori triggers DNA double-strand breaks and a DNA damage response in its host cells.

[12] Hanada K, Uchida T, Tsukamoto Y, Watada M, Yamaguchi N, Yamamoto K, Shiota S, Moriyama M, Graham DY, Yamaoka Y (2014). Helicobacter pylori infection introduces DNA double-strand breaks in host cells. Infection and immunity.

[13] Linzer DI, Levine AJ (1979). Characterization of a 54K dalton cellular SV40 tumor antigen present in SV40-transformed cells and uninfected embryonal carcinoma cells. Cell.

[14] Sakaguchi K, Herrera JE, Saito S, Miki T, Bustin M, Vassilev A, Anderson CW, Appella E (1998). DNA damage activates p53 through a phosphorylation-acetylation cascade. Genes & development.

[15] Tibbetts RS, Brumbaugh KM, Williams JM, Sarkaria JN, Cliby WA, Shieh SY, Taya Y, Prives C, Abraham RT (1999). A role for ATR in the DNA damage-induced phosphorylation of p53. Genes & development.

[16] Giaccia AJ, Kastan MB (1998). The complexity of p53 modulation: emerging patterns from divergent signals. Genes & development.

[17] Meng X, Franklin DA, Dong J, Zhang Y (2014). MDM2-p53 pathway in hepatocellular carcinoma. Cancer research.

[18] Chen D, Kon N, Li M, Zhang W, Qin J, Gu W (2005). ARF-BP1/Mule is a critical mediator of the ARF tumor suppressor. Cell.

[19] Sherr CJ, McCormick F (2002). The RB and p53 pathways in cancer. Cancer cell.

[20] Gartel AL, Serfas MS, Tyner AL (1996). p21—negative regulator of the cell cycle.

[21] Miyashita T, Reed JC (1995). Tumor suppressor p53 is a direct transcriptional activator of the human bax gene. Cell.

[22] Soussi T, Wiman KG (2007). Shaping genetic alterations in human cancer: the p53 mutation paradigm. Cancer cell.

[23] Kenzelmann Broz D, Attardi LD (2010). In vivo analysis of p53 tumor suppressor function using genetically engineered mouse models. Carcinogenesis.

[24] Zheng HC, Li XH, Hara T, Masuda S, Yang XH, Guan YF, Takano Y (2008). Mixed-type gastric carcinomas exhibit more aggressive features and indicate the histogenesis of carcinomas. Virchows Arch.

[25] Hu B, El Hajj N, Sittler S, Lammert N, Barnes R, Meloni-Ehrig A (2012). Gastric cancer: Classification, histology and application of molecular pathology. J Gastrointest Oncol.

[26] Correa P (1992). Human gastric carcinogenesis: a multistep and multifactorial process—First American Cancer Society Award Lecture on Cancer Epidemiology and Prevention. Cancer research.

[27] Satoh K, Kihira K, Kawata H, Tokumaru K, Kumakura Y, Ishino Y, Kawakami S, Inoue K, Kojima T, Satoh Y, Mutoh H, Sugano K (2001). p53 expression in the gastric mucosa before and after eradication of Helicobacter pylori. Helicobacter.

[28] Shimizu T, Marusawa H, Matsumoto Y, Inuzuka T, Ikeda A, Fujii Y, Minamiguchi S, Miyamoto S, Kou T, Sakai Y, Crabtree JE, Chiba T (2014). Accumulation of somatic mutations in TP53 in gastric epithelium with Helicobacter pylori infection. Gastroenterology.

[29] Teh M, Tan KB, Seet BL, Yeoh KG (2002). Study of p53 immunostaining in the gastric epithelium of cagA-positive and cagA-negative Helicobacter pylori gastritis. Cancer.

[30] Petersson F, Franzen LE, Borch K (2010). Characterization of the gastric cardia in volunteers from the general population. Type of mucosa, Helicobacter pylori infection, inflammation, mucosal proliferative activity, p53 and p21 expression, and relations to gastritis. Digestive diseases and sciences.

[31] Morales-Fuentes GA, Zarate-Osorno A, Quinonez-Urrego EE, Antonio-Manrique M, Martinez-Garcia CL, Figueroa-Barojas P, Zamorano-Orozco Y, Leal-Osuna SE, Martinez-Camacho C, Mejia-Cuan LA, Rivera-Nava CA, Sanchez-Chavez X, Ramirez-Ramirez MA (2013). [p53 expression in the gastric mucosa of patients infected with Helicobacter pylori]. Revista de gastroenterologia de Mexico.

[32] Yemelyanova A, Vang R, Kshirsagar M, Lu D, Marks MA, Shih Ie M, Kurman RJ (2011). Immunohistochemical staining patterns of p53 can serve as a surrogate marker for TP53 mutations in ovarian carcinoma: an immunohistochemical and nucleotide sequencing analysis. Modern pathology.

[33] Li JH, Shi XZ, Lv S, Liu M, Xu GW (2005). Effect of Helicobacter pylori infection on p53 expression of gastric mucosa and adenocarcinoma with microsatellite instability. World journal of gastroenterology.

[34] Salih BA, Gucin Z, Bayyurt N (2013). A study on the effect of Helicobacter pylori infection on p53 expression in gastric cancer and gastritis tissues. Journal of infection in developing countries.

[35] Kalo E, Kogan-Sakin I, Solomon H, Bar-Nathan E, Shay M, Shetzer Y, Dekel E, Goldfinger N, Buganim Y, Stambolsky P, Goldstein I, Madar S, Rotter V (2012). Mutant p53R273H attenuates the expression of phase 2 detoxifying enzymes and promotes the survival of cells with high levels of reactive oxygen species. Journal of cell science.

[36] Weissmueller S, Manchado E, Saborowski M, Morris JPt, Wagenblast E, Davis CA, Moon SH, Pfister NT, Tschaharganeh DF, Kitzing T, Aust D, Markert EK, Wu J, Grimmond SM, Pilarsky C, Prives C (2014). Mutant p53 drives pancreatic cancer metastasis through cell-autonomous PDGF receptor beta signaling. Cell.

[37] Liu DP, Song H, Xu Y (2010). A common gain of function of p53 cancer mutants in inducing genetic instability. Oncogene.

[38] Kato S, Han SY, Liu W, Otsuka K, Shibata H, Kanamaru R, Ishioka C (2003). Understanding the function-structure and function-mutation relationships of p53 tumor suppressor protein by high-resolution missense mutation analysis.

[39] Oren M, Rotter V (2010). Mutant p53 gain-of-function in cancer. Cold Spring Harbor perspectives in biology.

[40] Weisz L, Oren M, Rotter V (2007). Transcription regulation by mutant p53. Oncogene.

[41] Kanda M, Sadakari Y, Borges M, Topazian M, Farrell J, Syngal S, Lee J, Kamel I, Lennon AM, Knight S, Fujiwara S, Hruban RH, Canto MI, Goggins M (2013). Mutant TP53 in duodenal samples of pancreatic juice from patients with pancreatic cancer or high-grade dysplasia. Clinical gastroenterology and hepatology.

[42] Busuttil RA, Zapparoli GV, Haupt S, Fennell C, Wong SQ, Pang JM, Takeno EA, Mitchell C, Di Costanzo N, Fox S, Haupt Y, Dobrovic A, Boussioutas A (2014). Role of p53 in the progression of gastric cancer. Oncotarget.

[43] Zheng Y, Wang L, Zhang JP, Yang JY, Zhao ZM, Zhang XY (2010). Expression of p53, c-erbB-2 and Ki67 in intestinal metaplasia and gastric carcinoma. World journal of gastroenterology.

[44] Lopez-Saez JB, Gomez-Biondi V, Santamaria-Rodriguez G, Dominguez-Villar M, Amaya-Vidal A, Lorenzo-Penuelas A, Senra-Varela A (2010). Concurrent overexpression of serum p53 mutation related with Helicobacter pylori infection. Journal of experimental & clinical cancer research.

[45] Derks S, Bass AJ (2014). Mutational signatures in Helicobacter pylori-induced gastric cancer: lessons from new sequencing technologies. Gastroenterology.

[46] Khromova NV, Kopnin PB, Stepanova EV, Agapova LS, Kopnin BP (2009). p53 hot-spot mutants increase tumor vascularization via ROS-mediated activation of the HIF1/VEGF-A pathway. Cancer letters.

[47] Palli D, Caporaso NE, Shiao YH, Saieva C, Amorosi A, Masala G, Rice JM, Fraumeni JF (1997). Diet, Helicobacter pylori, and p53 mutations in gastric cancer: a molecular epidemiology study in Italy. Cancer epidemiology, biomarkers & prevention.

[48] Shibata A, Parsonnet J, Longacre TA, Garcia MI, Puligandla B, Davis RE, Vogelman JH, Orentreich N, Habel LA (2002). CagA status of Helicobacter pylori infection and p53 gene mutations in gastric adenocarcinoma. Carcinogenesis.

[49] Oda T, Murakami K, Nishizono A, Kodama M, Nasu M, Fujioka T (2002). Long-term Helicobacter pylori infection in Japanese monkeys induces atrophic gastritis and accumulation of mutations in the p53 tumor suppressor gene. Helicobacter.

[50] Murakami K, Fujioka T, Kodama M, Honda S, Okimoto T, Oda T, Nishizono A, Sato R, Kubota T, Kagawa J, Nasu M (2002). Analysis of p53 mutations and Helicobacter pylori infection in human and animal models. Journal of gastroenterology.

[51] Saxena A, Shukla SK, Prasad KN, Ghoshal UC (2012). Analysis of p53, K-ras gene mutation & Helicobacter pylori infection in patients with gastric cancer & peptic ulcer disease at a tertiary care hospital in north India. The Indian journal of medical research.

[52] Matsumoto Y, Marusawa H, Kinoshita K, Endo Y, Kou T, Morisawa T, Azuma T, Okazaki IM, Honjo T, Chiba T (2007). Helicobacter pylori infection triggers aberrant expression of activation-induced cytidine deaminase in gastric epithelium. Nature medicine.

[53] Goncalves ML, Borja SM, Cordeiro JA, Saddi VA, Ayres FM, Vilanova-Costa CA, Silva AM (2014). Association of the TP53 codon 72 polymorphism and breast cancer risk: a meta-analysis. SpringerPlus.

[54] Wang S, Lan X, Tan S, Wang S, Li Y (2013). P53 codon 72 Arg/Pro polymorphism and lung cancer risk in Asians: an updated meta-analysis. Tumour biology.

[55] Gemignani F, Moreno V, Landi S, Moullan N, Chabrier A, Gutierrez-Enriquez S, Hall J, Guino E, Peinado MA, Capella G, Canzian F (2004). A TP53 polymorphism is associated with increased risk of colorectal cancer and with reduced levels of TP53 mRNA. Oncogene.

[56] Hiyama T, Tanaka S, Kitadai Y, Ito M, Sumii M, Yoshihara M, Shimamoto F, Haruma K, Chayama K (2002). p53 Codon 72 polymorphism in gastric cancer susceptibility in patients with Helicobacter pylori-associated chronic gastritis. International journal of cancer.

[57] Ke-Xiang Z, Yu-Min L, Xun L, Wen-Ce Z, Yong S, Tao L (2012). Study on the association of p53 codon 72 polymorphisms with risk of gastric cancer in high incidence Hexi area of Gansu Province in China. Molecular biology reports.

[58] Pandey R, Misra V, Misra SP, Dwivedi M, Misra A (2014). Helicobacter pylori infection and a P53 codon 72 single nucleotide polymorphism: a reason for an unexplained Asian enigma. Asian Pacific journal of cancer prevention.

[59] Bochkareva E, Kaustov L, Ayed A, Yi GS, Lu Y, Pineda-Lucena A, Liao JC, Okorokov AL, Milner J, Arrowsmith CH, Bochkarev A (2005). Single-stranded DNA mimicry in the p53 transactivation domain interaction with replication protein A.

[60] Koeppel M, Garcia-Alcalde F, Glowinski F, Schlaermann P, Meyer TF (2015). Helicobacter pylori infection causes characteristic DNA damage patterns in human cells. Cell reports.

[61] Zannini L, Delia D, Buscemi G (2014). CHK2 kinase in the DNA damage response and beyond. Journal of molecular cell biology.

[62] Reinhardt HC, Aslanian AS, Lees JA, Yaffe MB (2007). p53-deficient cells rely on ATM- and ATR-mediated checkpoint signaling through the p38MAPK/MK2 pathway for survival after DNA damage. Cancer cell.

[63] Gao K, Wang C, Jin X, Xiao J, Zhang E, Yang X, Wang D, Huang H, Yu L, Zhang P (2016). RNF12 promotes p53-dependent cell growth suppression and apoptosis by targeting MDM2 for destruction. Cancer letters.

[64] Joseph TW, Zaika A, Moll UM (2003). Nuclear and cytoplasmic degradation of endogenous p53 and HDM2 occurs during down-regulation of the p53 response after multiple types of DNA damage. FASEB journal.

[65] Moll UM, Petrenko O (2003). The MDM2-p53 interaction. Molecular cancer research : MCR.

[66] Yu Q, Li Y, Mu K, Li Z, Meng Q, Wu X, Wang Y, Li L (2014). Amplification of Mdmx and overexpression of MDM2 contribute to mammary carcinogenesis by substituting for p53 mutations. Diagnostic pathology.

[67] Sehdev V, Katsha A, Arras J, Peng D, Soutto M, Ecsedy J, Zaika A, Belkhiri A, El-Rifai W (2014). HDM2 regulation by AURKA promotes cell survival in gastric cancer. Clinical cancer research.

[68] Yong X, Tang B, Li BS, Xie R, Hu CJ, Luo G, Qin Y, Dong H, Yang SM (2015). Helicobacter pylori virulence factor CagA promotes tumorigenesis of gastric cancer via multiple signaling pathways. Cell communication and signaling.

[69] Zaika AI, Wei J, Noto JM, Peek RM (2015). Microbial Regulation of p53 Tumor Suppressor. PLoS pathogens.

[70] Kodama M, Fujioka T, Murakami K, Okimoto T, Sato R, Watanabe K, Nasu M (2005). Eradication of Helicobacter pylori reduced the immunohistochemical detection of p53 and MDM2 in gastric mucosa. Journal of gastroenterology and hepatology.

[71] Nakajima N, Ito Y, Yokoyama K, Uno A, Kinukawa N, Nemoto N, Moriyama M (2009). The Expression of Murine Double Minute 2 (MDM2) on Helicobacter pylori-Infected Intestinal Metaplasia and Gastric Cancer. Journal of clinical biochemistry and nutrition.

[72] Hayakawa Y, Fox JG, Gonda T, Worthley DL, Muthupalani S, Wang TC (2013). Mouse models of gastric cancer. Cancers.

[73] Wei J, Nagy TA, Vilgelm A, Zaika E, Ogden SR, Romero-Gallo J, Piazuelo MB, Correa P, Washington MK, El-Rifai W, Peek RM, Zaika A (2010). Regulation of p53 tumor suppressor by Helicobacter pylori in gastric epithelial cells. Gastroenterology.

[74] Ogawara Y, Kishishita S, Obata T, Isazawa Y, Suzuki T, Tanaka K, Masuyama N, Gotoh Y (2002). Akt enhances Mdm2-mediated ubiquitination and degradation of p53. The Journal of biological chemistry.

[75] Fenouille N, Puissant A, Tichet M, Zimniak G, Abbe P, Mallavialle A, Rocchi S, Ortonne JP, Deckert M, Ballotti R, Tartare-Deckert S (2011). SPARC functions as an anti-stress factor by inactivating p53 through Akt-mediated MDM2 phosphorylation to promote melanoma cell survival. Oncogene.

[76] Zaika AI (2015). Bacterial pathogen Helicobacter pylori: A bad AKTor inhibits p53 protein activity [corrected]. Digestive diseases and sciences.

[77] Shu X, Yang Z, Li ZH, Chen L, Zhou XD, Xie Y, Lu NH (2015). Helicobacter pylori infection activates the Akt-Mdm2-p53 signaling pathway in gastric epithelial cells. Digestive diseases and sciences.

[78] Phillips A, Jones CJ, Blaydes JP (2006). The mechanisms of regulation of Hdm2 protein level by serum growth factors. FEBS letters.

[79] Sato A, Sunayama J, Matsuda K, Seino S, Suzuki K, Watanabe E, Tachibana K, Tomiyama A, Kayama T, Kitanaka C (2011). MEK-ERK signaling dictates DNA-repair gene MGMT expression and temozolomide resistance of stem-like glioblastoma cells via the MDM2-p53 axis. Stem cells.

[80] Bhardwaj V, Noto JM, Wei J, Andl C, El-Rifai W, Peek RM, Zaika AI (2015). Helicobacter pylori bacteria alter the p53 stress response via ERK-HDM2 pathway. Oncotarget.

[81] Shi D, Gu W (2012). Dual Roles of MDM2 in the Regulation of p53: Ubiquitination Dependent and Ubiquitination Independent Mechanisms of MDM2 Repression of p53 Activity. Genes cancer.

[82] Wang J, Ding S, Duan Z, Xie Q, Zhang T, Zhang X, Wang Y, Chen X, Zhuang H, Lu F (2015). Role of p14ARF-HDM2-p53 axis in SOX6-mediated tumor suppression. Oncogene.

[83] Wei J, Noto JM, Zaika E, Romero-Gallo J, Piazuelo MB, Schneider B, El-Rifai W, Correa P, Peek RM, Zaika AI (2015). Bacterial CagA protein induces degradation of p53 protein in a p14ARF-dependent manner. Gut.

[84] Iida S, Akiyama Y, Nakajima T, Ichikawa W, Nihei Z, Sugihara K, Yuasa Y (2000). Alterations and hypermethylation of the p14(ARF) gene in gastric cancer. International journal of cancer.

[85] Venkatanarayan A, Raulji P, Norton W, Flores ER (2016). Novel therapeutic interventions for p53-altered tumors through manipulation of its family members, p63 and p73. Cell cycle (Georgetown, Tex).

[86] Moll UM, Slade N (2004). p63 and p73: roles in development and tumor formation. Molecular cancer research.

[87] Bourdon JC (2007). p53 and its isoforms in cancer. British journal of cancer.

[88] Wei J, Zaika E, Zaika A (2012). p53 Family: role of protein isoforms in human cancer. Journal of nucleic acids.

[89] Wei J, O'Brien D, Vilgelm A, Piazuelo MB, Correa P, Washington MK, El-Rifai W, Peek RM, Zaika A (2008). Interaction of Helicobacter pylori with gastric epithelial cells is mediated by the p53 protein family. Gastroenterology.

[90] Khoury MP, Bourdon JC (2011). p53 Isoforms: An Intracellular Microprocessor?. Genes cancer.

[91] Nutthasirikul N, Limpaiboon T, Leelayuwat C, Patrakitkomjorn S, Jearanaikoon P (2013). Ratio disruption of the 133p53 and TAp53 isoform equilibrium correlates with poor clinical outcome in intrahepatic cholangiocarcinoma. International journal of oncology.

[92] Slatter TL, Hung N, Bowie S, Campbell H, Rubio C, Speidel D, Wilson M, Baird M, Royds JA, Braithwaite AW (2015). Delta122p53, a mouse model of Delta133p53alpha, enhances the tumor-suppressor activities of an attenuated p53 mutant. Cell death & disease.

[93] Bossis G, Malnou CE, Farras R, Andermarcher E, Hipskind R, Rodriguez M, Schmidt D, Muller S, Jariel-Encontre I, Piechaczyk M (2005). Down-regulation of c-Fos/c-Jun AP-1 dimer activity by sumoylation. Molecular and cellular biology.

[94] Wei J, Noto J, Zaika E, Romero-Gallo J, Correa P, El-Rifai W, Peek RM, Zaika A (2012). Pathogenic bacterium Helicobacter pylori alters the expression profile of p53 protein isoforms and p53 response to cellular stresses.

[95] Zhao XD, Lu YY, Guo H, Xie HH, He LJ, Shen GF, Zhou JF, Li T, Hu SJ, Zhou L, Han YN, Liang SL, Wang X, Wu KC, Shi YQ, Nie YZ (2015). MicroRNA-7/NF-kappaB signaling regulatory feedback circuit regulates gastric carcinogenesis. The Journal of cell biology.

[96] Schumacher B, Mondry J, Thiel P, Weyand M, Ottmann C (2010). Structure of the p53 C-terminus bound to 14-3-3: implications for stabilization of the p53 tetramer. FEBS letters.

[97] Nagappan A, Park HS, Park KI, Hong GE, Yumnam S, Lee HJ, Kim MK, Kim EH, Lee WS, Lee WJ, Cho MJ, Lee WK, Won CK, Cho JH, Kim GS (2013). Helicobacter pylori infection combined with DENA revealed altered expression of p53 and 14-3-3 isoforms in Gulo−/− mice. Chemico-biological interactions.

[98] Sullivan A, Lu X (2007). ASPP: a new family of oncogenes and tumour suppressor genes. British journal of cancer.

[99] Wang Y, Godin-Heymann N, Dan Wang X, Bergamaschi D, Llanos S, Lu X (2013). ASPP1 and ASPP2 bind active RAS, potentiate RAS signalling and enhance p53 activity in cancer cells. Cell death and differentiation.

[100] Notari M, Hu Y, Koch S, Lu M, Ratnayaka I, Zhong S, Baer C, Pagotto A, Goldin R, Salter V, Candi E, Melino G, Lu X (2011). Inhibitor of apoptosis-stimulating protein of p53 (iASPP) prevents senescence and is required for epithelial stratification.

[101] Meng WD, Chu RX, Wang BZ, Wang LP, Ma LL, Wang LX (2013). Helicobacter pylori infection and expressions of apoptosis-related proteins p53, ASPP2 and iASPP in gastric cancer and precancerous lesions. Pathol Biol.

[102] Reingewertz TH, Iosub-Amir A, Bonsor DA, Mayer G, Amartely H, Friedler A, Sundberg EJ (2015). An Intrinsically Disordered Region in the Proapoptotic ASPP2 Protein Binds to the Helicobacter pylori Oncoprotein CagA. Biochemistry.

[103] Buti L, Spooner E, Van der Veen AG, Rappuoli R, Covacci A, Ploegh HL (2011). Helicobacter pylori cytotoxin-associated gene A (CagA) subverts the apoptosis-stimulating protein of p53 (ASPP2) tumor suppressor pathway of the host.

[104] Nesic D, Buti L, Lu X, Stebbins CE (2014). Structure of the Helicobacter pylori CagA oncoprotein bound to the human tumor suppressor ASPP2.

[105] Hao W, Yuan X, Yu L, Gao C, Sun X, Wang D, Zheng Q (2015). Licochalcone A-induced human gastric cancer BGC-823 cells apoptosis by regulating ROS-mediated MAPKs and PI3K/AKT signaling pathways. Scientific reports.

